# Supplementary material for: Unveiling the Bioactive Potential of the Invasive Jellyfish Phyllorhiza punctata Through Integrative Transcriptomic and Proteomic Analyses
Source: Biomolecules. 2025 Aug 4;15(8):1121. doi: 10.3390/biom15081121 (PMC12383608; doi:10.3390/biom15081121)
Supplement: Supplementary file 1 [file biomolecules-15-01121-s001.zip › Suplementary Material/FileS3/PEPFOLD4/XP_065051222_9675/clusters.html]

 PEP-FOLD clusters


Download archive of all models  here   

|  |  |  |
| --- | --- | --- |
| # | file | sOPEP |
| # cluster 1 - Size: 16 |  |  |
|  | XP\_065051222\_9675-model1 | -7.65709 |
|  | XP\_065051222\_9675-model1.1 | -7.65709 |
|  | XP\_065051222\_9675-model1.2 | -7.65709 |
|  | XP\_065051222\_9675-model1.3 | -7.65709 |
|  | XP\_065051222\_9675-model1.4 | -7.65709 |
|  | XP\_065051222\_9675-model1.5 | -7.65709 |
|  | XP\_065051222\_9675-model1.6 | -7.65709 |
|  | XP\_065051222\_9675-model1.7 | -7.65709 |
|  | XP\_065051222\_9675-model1.8 | -7.65709 |
|  | XP\_065051222\_9675-model1.9 | -7.65709 |
|  | XP\_065051222\_9675-model1.10 | -7.65709 |
|  | XP\_065051222\_9675-model1.11 | -7.65709 |
|  | XP\_065051222\_9675-model1.12 | -7.65709 |
|  | XP\_065051222\_9675-model1.13 | -7.65709 |
|  | XP\_065051222\_9675-model1.14 | -7.65709 |
|  | XP\_065051222\_9675-model1.15 | -7.65709 |
| # cluster 2 - Size: 12 |  |  |
|  | XP\_065051222\_9675-model2 | -7.65709 |
|  | XP\_065051222\_9675-model2.1 | -7.65709 |
|  | XP\_065051222\_9675-model2.2 | -7.65709 |
|  | XP\_065051222\_9675-model2.3 | -7.65709 |
|  | XP\_065051222\_9675-model2.4 | -7.65709 |
|  | XP\_065051222\_9675-model2.5 | -7.65709 |
|  | XP\_065051222\_9675-model2.6 | -7.65709 |
|  | XP\_065051222\_9675-model2.7 | -7.65709 |
|  | XP\_065051222\_9675-model2.8 | -7.65709 |
|  | XP\_065051222\_9675-model2.9 | -7.65709 |
|  | XP\_065051222\_9675-model2.10 | -7.65709 |
|  | XP\_065051222\_9675-model2.11 | -7.65709 |
| # cluster 3 - Size: 12 |  |  |
|  | XP\_065051222\_9675-model3 | -7.65709 |
|  | XP\_065051222\_9675-model3.1 | -7.65709 |
|  | XP\_065051222\_9675-model3.2 | -7.65709 |
|  | XP\_065051222\_9675-model3.3 | -7.65709 |
|  | XP\_065051222\_9675-model3.4 | -7.65709 |
|  | XP\_065051222\_9675-model3.5 | -7.65709 |
|  | XP\_065051222\_9675-model3.6 | -7.65709 |
|  | XP\_065051222\_9675-model3.7 | -7.65709 |
|  | XP\_065051222\_9675-model3.8 | -7.65709 |
|  | XP\_065051222\_9675-model3.9 | -7.65709 |
|  | XP\_065051222\_9675-model3.10 | -7.65709 |
|  | XP\_065051222\_9675-model3.11 | -7.65709 |
| # cluster 4 - Size: 29 |  |  |
|  | XP\_065051222\_9675-model4 | -7.65709 |
|  | XP\_065051222\_9675-model4.1 | -7.65709 |
|  | XP\_065051222\_9675-model4.2 | -7.65709 |
|  | XP\_065051222\_9675-model4.3 | -7.65709 |
|  | XP\_065051222\_9675-model4.4 | -7.65709 |
|  | XP\_065051222\_9675-model4.5 | -7.65709 |
|  | XP\_065051222\_9675-model4.6 | -7.65709 |
|  | XP\_065051222\_9675-model4.7 | -7.65709 |
|  | XP\_065051222\_9675-model4.8 | -7.65709 |
|  | XP\_065051222\_9675-model4.9 | -7.65709 |
|  | XP\_065051222\_9675-model4.10 | -7.65709 |
|  | XP\_065051222\_9675-model4.11 | -7.65709 |
|  | XP\_065051222\_9675-model4.12 | -7.65709 |
|  | XP\_065051222\_9675-model4.13 | -7.65709 |
|  | XP\_065051222\_9675-model4.14 | -7.65709 |
|  | XP\_065051222\_9675-model4.15 | -7.65709 |
|  | XP\_065051222\_9675-model4.16 | -7.65709 |
|  | XP\_065051222\_9675-model4.17 | -7.65709 |
|  | XP\_065051222\_9675-model4.18 | -7.65709 |
|  | XP\_065051222\_9675-model4.19 | -7.65709 |
|  | XP\_065051222\_9675-model4.20 | -7.65709 |
|  | XP\_065051222\_9675-model4.21 | -7.65709 |
|  | XP\_065051222\_9675-model4.22 | -7.65709 |
|  | XP\_065051222\_9675-model4.23 | -7.65709 |
|  | XP\_065051222\_9675-model4.24 | -7.65709 |
|  | XP\_065051222\_9675-model4.25 | -7.65709 |
|  | XP\_065051222\_9675-model4.26 | -7.65709 |
|  | XP\_065051222\_9675-model4.27 | -7.65709 |
|  | XP\_065051222\_9675-model4.28 | -7.65709 |
| # cluster 5 - Size: 9 |  |  |
|  | XP\_065051222\_9675-model5 | -7.65709 |
|  | XP\_065051222\_9675-model5.1 | -7.65709 |
|  | XP\_065051222\_9675-model5.2 | -7.65709 |
|  | XP\_065051222\_9675-model5.3 | -7.65709 |
|  | XP\_065051222\_9675-model5.4 | -7.65709 |
|  | XP\_065051222\_9675-model5.5 | -7.65709 |
|  | XP\_065051222\_9675-model5.6 | -7.65709 |
|  | XP\_065051222\_9675-model5.7 | -7.65709 |
|  | XP\_065051222\_9675-model5.8 | -7.65709 |
| # cluster 6 - Size: 5 |  |  |
|  | XP\_065051222\_9675-model6 | -7.65709 |
|  | XP\_065051222\_9675-model6.1 | -7.65709 |
|  | XP\_065051222\_9675-model6.2 | -7.65709 |
|  | XP\_065051222\_9675-model6.3 | -7.65709 |
|  | XP\_065051222\_9675-model6.4 | -7.65709 |
| # cluster 7 - Size: 9 |  |  |
|  | XP\_065051222\_9675-model7 | -7.65709 |
|  | XP\_065051222\_9675-model7.1 | -7.65709 |
|  | XP\_065051222\_9675-model7.2 | -7.65709 |
|  | XP\_065051222\_9675-model7.3 | -7.65709 |
|  | XP\_065051222\_9675-model7.4 | -7.65709 |
|  | XP\_065051222\_9675-model7.5 | -7.65709 |
|  | XP\_065051222\_9675-model7.6 | -7.65709 |
|  | XP\_065051222\_9675-model7.7 | -7.65709 |
|  | XP\_065051222\_9675-model7.8 | -7.65709 |
| # cluster 8 - Size: 4 |  |  |
|  | XP\_065051222\_9675-model8 | -7.65709 |
|  | XP\_065051222\_9675-model8.1 | -7.65709 |
|  | XP\_065051222\_9675-model8.2 | -7.65709 |
|  | XP\_065051222\_9675-model8.3 | -7.65709 |
| # cluster 9 - Size: 3 |  |  |
|  | XP\_065051222\_9675-model9 | -7.65709 |
|  | XP\_065051222\_9675-model9.1 | -7.65709 |
|  | XP\_065051222\_9675-model9.2 | -7.65709 |
| # cluster 10 - Size: 1 |  |  |
|  | XP\_065051222\_9675-model10 | -7.65709 |
|  |  |  |
